# Currently available and experimental dyes for intraoperative near-infrared fluorescence imaging of the ureters: a systematic review

**DOI:** 10.1007/s10151-019-01973-4

**Published:** 2019-04-27

**Authors:** M. D. Slooter, A. Janssen, W. A. Bemelman, P. J. Tanis, R. Hompes

**Affiliations:** Department of Surgery, Amsterdam UMC, University of Amsterdam, G4, Postbox 22660, 1100 DD Amsterdam, The Netherlands

**Keywords:** Ureter detection, Fluorescence, Near-infrared, ICG, MB, Iatrogenic ureteral injury

## Abstract

**Background:**

Iatrogenic ureteral injury (IUI) following abdominal surgery has a relatively low incidence, but is associated with high risks of morbidity and mortality. Conventional assessment of IUI includes visual inspection and palpation. This is especially challenging during laparoscopic procedures and has translated into an increased risk of IUI. The use of near-infrared fluorescent (NIRF) imaging is currently being considered as a novel method to identify the ureters intraoperatively. The aim of this review is to describe the currently available and experimental dyes for ureter visualization and to evaluate their feasibility of using them and their effectiveness.

**Methods:**

This article adhered to the Preferred Reporting Items for Systematic Reviews and Meta-Analyses (PRISMA) standard for systematic reviews. A systematic literature search was performed in the PubMed database. All included articles were screened for eligibility by two authors. Three clinical trial databases were consulted to identify ongoing or completed unpublished trials. Risk of bias was assessed for all articles.

**Results:**

The search yielded 20 articles on ureter visualization. Two clinically available dyes, indocyanine green (ICG) and methylene blue (MB), and eight experimental dyes were described and assessed for their feasibility to identify the ureter. Two ongoing clinical trials on CW800-BK and one trial on ZW800-1 for ureter visualization were identified.

**Conclusions:**

Currently available dyes, ICG and MB, are safe, but suboptimal for ureter visualization based on the route of administration and optical properties, respectively. Currently, MB has potential to be routinely used for ureter visualization in most patients, but (cRGD-)ZW800-1 holds potential for this role in the future, owing to its exclusive renal clearance and the near absence of background. To assess the benefit of NIRF imaging for reducing the incidence of IUI, larger patient cohorts need to be examined.

**Electronic supplementary material:**

The online version of this article (10.1007/s10151-019-01973-4) contains supplementary material, which is available to authorized users.

## Introduction

Iatrogenic ureteral injury (IUI) is a serious surgical complication, with a reported overall incidence up to 1.2% [1, 2]. The highest prevalence of IUI is recorded in gynaecological procedures (50% of IUIs), followed by urologic (30%), and colorectal surgery (5–15%) [3]. Unfortunately, most IUIs are identified post-operatively [4]. If they were detected intraoperatively, this would allow for immediate repair and improved outcomes [5].

Intraoperative ureter identification is most often achieved by visual inspection and palpation. Both can be more challenging during laparoscopic procedures, translating into a higher risk of IUI [6, 7]. An alternative and effective method to identify IUI is cystoscopy, whereby the ureteral jets are visualized using a urine colouring dye [8]. However, the most studied dye, indigo carmine, has been discontinued and no consensus has been reached on a suitable replacement [9].

**Fig. 1:**
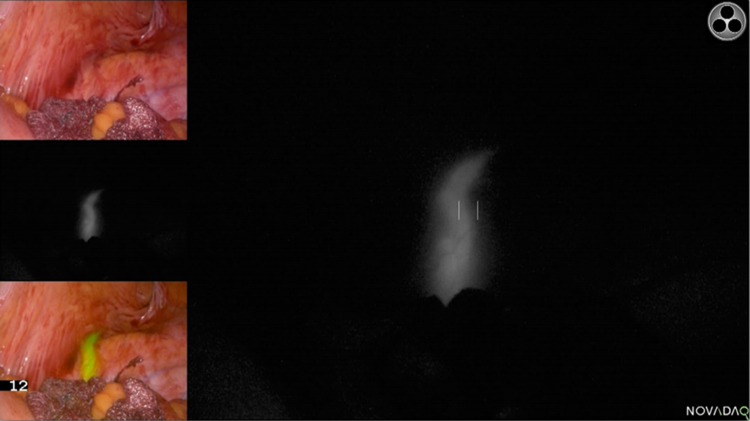
Visualization of the ureter using methylene blue during open surgery. Reproduced with permission from Barnes et al. 2018 [21] (CC BY 4.0)

An emerging field is the use of near-infrared fluorescence (NIRF) imaging. NIRF is a promising technique for the intraoperative visualization of tumours, sentinel lymph nodes, tissue perfusion, and vital structures [10]. This provides the surgeon an enhanced reality beyond standard white light visual inspection and palpation, as a fluorescent signal from the structure of interest can be observed using real-time imaging (Fig. 1). NIRF dyes emit light outside of the visible spectrum and present little tissue autofluorescence, making them optimal for imaging during surgical procedures. Only a few studies have investigated NIRF imaging for intraoperative ureter detection.

This review focuses on the current literature describing the use of fluorescent dyes to identify the ureters during surgery. Our primary goal is to provide an overview of experimental and clinically available dyes. In addition, we aim to identify clinical trials that are being performed using these experimental dyes.

## Materials and methods

### Article inclusion

This systematic review was conducted in accordance with the guidelines from the Preferred Reporting Items for Systematic reviews and Meta-Analysis (PRISMA) group [11]. A comprehensive literature search was performed using a combination of free-text terms and controlled vocabulary in the PubMed and Embase database. Search terms included keywords to cover the subjects of fluorescent imaging, ureter visualization, and intraoperative use. An overview of our search terms can be found in supplementary Table 1. The reference lists of included articles were scanned to potentially obtain additional articles.

**Table 1:** Characteristics of fluorescent dyes that have been used for visualization of the ureter. Data are adapted from Gioux et al. [41]

	Dye	*E*_x_ (nm)	*E*_m_ (nm)	Renal clearance	Ext coeff (M^−1^ cm^−1^)	Quantum yield (%)
Currently available dyes	ICG	807	822	–	121,000	9.3
	MB	670	690	+	71,200	3.8
Experimental dyes	CW800-CA	786	800	+	237,000	14.2
	CW800-BK [25]	774	790	NR	NR	NR
	ZW800-1	772	788	++	249,000	15.1
	cRGD-ZW800-1	NR	NR	++	NR	NR
	Fluorescein [42]	494	512	+	92,300	95.0
	Liposomal ICG	NR	NR	+	NR	NR
	Genhance 750	750	775	NR	240,000	NR
	UL-766 [37]	766	789	++	229,000	9.5
	UreterGlow [38]	800	830	+	NR	NR

– not renally cleared, + renally cleared by mainly hepatic, ++ (near) exclusive renal clearance, *E*_*x*_ excitation wavelength, *E*_*m*_ emission wavelength, *Ext Coeff* extinction coefficient, *NR* not reported, *ICG* indocyanine green, *MB* methylene blue

Final article inclusion was based on a three-phase process: the initial search in the databases, screening of the search results, and evaluation of the full-text articles based on our eligibility criteria. We conducted our database search on 16 November 2018 and included articles in any state of publication and from any year. Two authors (AJ and MS) independently screened the titles and abstracts of the initial search results using Covidence and based inclusion or exclusion on predetermined criteria (Supp. Table 2). After all articles were screened, conflicts on the eventual in- or exclusion of a paper were discussed in order to reach consensus. We then read the full-text articles of the screened list to determine eligibility for final inclusion. In order to be included in the analysis, manuscripts had to be full-text, presenting original data on at least one of the outcomes of interest: ureteral visualization, safety, feasibility, or dose-finding results in humans or animals.

Data were collected from each article and grouped per dye. The primary outcome of interest was the number of ureters identified. Secondary outcomes were post-operative complications and IUI incidence. Other outcomes included the duration of fluorescence, safety outcomes, and the signal-to-background ratio (SBR). The SBR is the ratio of the fluorescence signal of the ureter to the background signal of the surrounding tissue, and a SBR above 2 was considered clinically relevant [12].

### Quality assessment

Risk of bias was assessed using the Newcastle–Ottawa scale for cohort studies, and the SYRCLE risk of bias tool for the assessment of animal studies. Two researchers (AJ and MS) performed the quality assessment, and conflicts were discussed in order to reach consensus. For studies assessed with the Newcastle–Ottawa scale without a control group, the comparability domain was granted a maximum of one point if the exposed group was described sufficiently. Assessment of the adequacy of follow-up was performed with our primary outcome in mind, determining if the duration of imaging was long enough to expect the fluorescent signal to appear in the ureter. The SYRCLE risk of bias assessment was performed as described by Hooijmans et al. [13].

### Clinical trial search

A search in clinical trial databases was conducted to identify clinical trials testing novel dyes in any surgical setting for ureter delineation. To this end, the Clinicaltrails.gov database, ISRCTN registry, and EU Clinical trials register were consulted. The search was simplified by searching for ‘surgery’ and/or ‘ureter’. Completed trials that were not yet published and any ongoing trial were included. The results from the initial search were screened by one researcher (AJ).

## Results

**Fig. 2:**
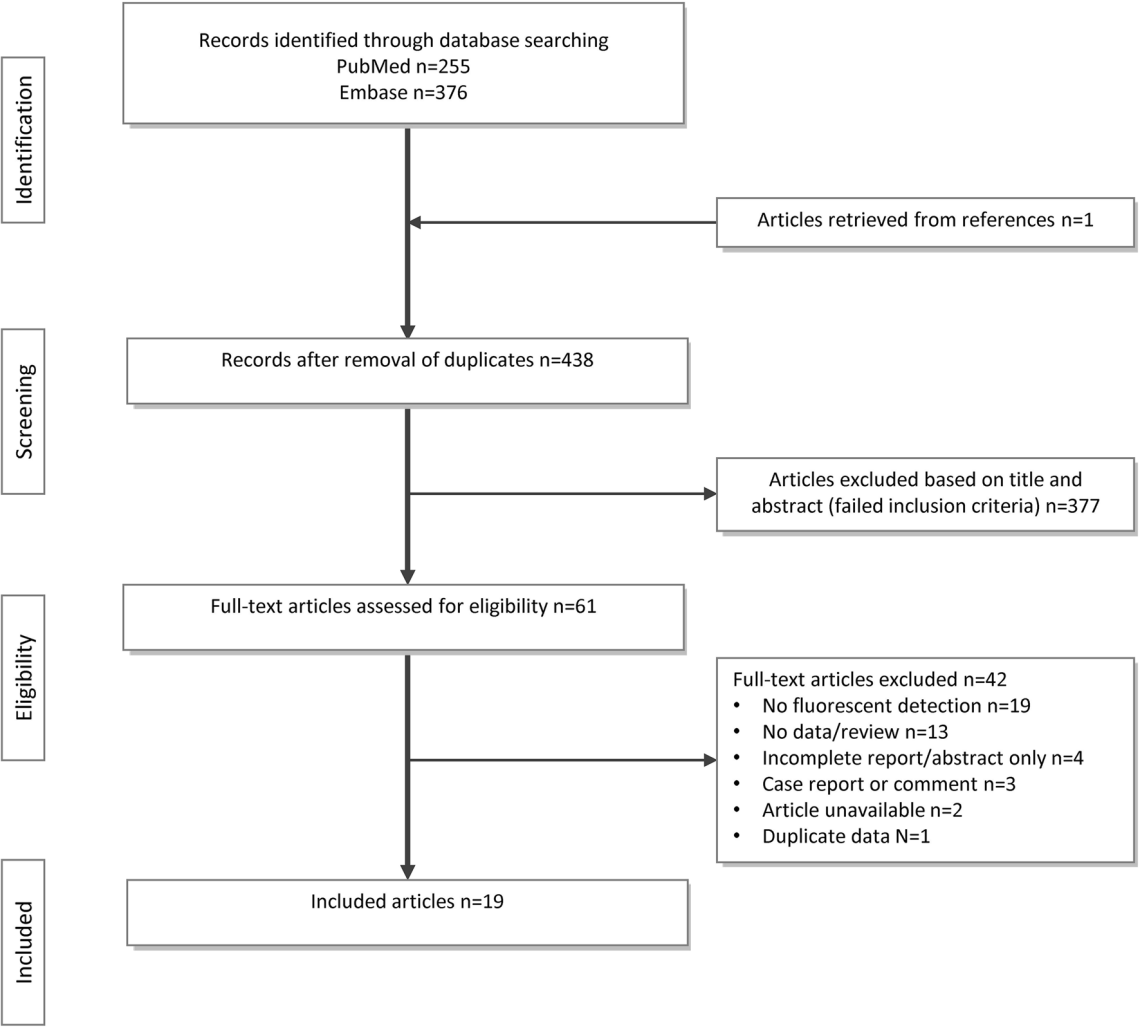
Overview of the number of articles in- or excluded

In total, 19 studies reporting on the use of ten fluorescent dyes met the selection criteria and were included in the systematic review. A PRISMA flow diagram showing article inclusion is presented in Fig. 2.

For the purpose of this review, the dyes have been divided into two sections: currently available dyes and experimental dyes. An overview of all dyes, including dye characteristics, is shown in Table 1.

### Currently available dyes

#### Indocyanine green

Two articles described the feasibility of indocyanine green (ICG) for ureter visualization in small patient cohorts (Table 2) [14, 15]. The quality of these articles was poor (*n* = 1) to fair (*n* = 1) (Supp. Table 3a). Lee et al. [16] were excluded as a duplicate from this review, because Lee et al. [15] include the patients cohort of seven patients described in the earlier article. Siddighi et al. described the use of ICG for ureter detection during laparoscopic sacrocolpopexy [14], and Lee et al. during robot-assisted ureteral reconstructions for various ureteral pathologies [15]. In these studies, the method of administration was retrograde injection of ICG into the ureter through ureteral catheters. The catheter was clamped after injection of ICG and allowed immediate visualization of all relevant ureters for the duration of the case. None of the studies specified the total number of identified ureters nor was the SBR measured. Nonetheless, the studies report precise identification, as background noise was absent due to the intraureteral injection of ICG [15]. Only one complication was noted and was due to trocar placement [15]. None of the patients required re-operation during follow-up. No adverse events were reported related to the use of ICG or catheter placement.

**Table 2:** Patient outcomes of human cohort studies using ICG and MB. Only patients where visualization using fluorescence was attempted are included in the table

	Article	Reference	Surgical specialism	Laparoscopic/open	Number of patients	Administration	Dose	Ureters identified	Complications
Indocyanine green	Siddighi et al. (2014)	[14]	Gynaecologic	Laparoscopic	> 10	Ureteral catheter	25 mg in 10 mL/ureter	All	None
	Lee et al. (2015)	[15]	Urologic	Laparoscopic	25	Ureteral catheter and/or PNT	25 mg in 10 mL/ureter	All	Yes, in one patient^a^
Methylene blue	Verbeek et al. (2013)	[18]	Abdominal	Open	12	Intravenous	0.25–1 mg/kg	24/24	None
	Yeung et al. (2016)	[19]	Colorectal	Both	8	Intravenous	0.25–1 mg/kg	10/11	None
	Al-Taher et al. (2016)	[20]	Colorectal	Laparoscopic	9	Intravenous	0.125–1.0 mg/kg	6/9	None
	Barnes et al. (2018)	[21]	Colorectal	Both	40	Intravenous	0.25–1 mg/kg	63/69	None

*NT* percutaneous nephrostomy tube, *ICG* indocyanine green, *MB* methylene blue

^a^Organ laceration due to trocar placement during uretero-ureterostomy

#### Methylene blue

Four good quality human studies and one animal study were identified (Table 2, Supp. Table 3 and 4) [17–21]. Matsui et al. reported visualization of the ureters in pigs after intravenous (IV) injection of methylene blue (MB), prompting clinical translation by others [18–21]. In humans, feasibility and dose-finding studies described MB use in open and laparoscopic abdominal procedures [18–21]. MB was injected IV with a dose between 0.25 and 1.0 mg/kg and allowed ureteral visualization after 10–15 min [21]. Duration of the staining was found to be up to 2-h post-injection, but this varied between patients [18, 21]. Verbeek et al. reported the possibility of visualizing all ureters [18], whereas the other studies identified 89% across all doses [19–21]. One-fifth of detected ureters was only identified using fluorescence, indicating the potential additional clinical benefit. The studies found mean SBRs higher than 2, with the highest recorded SBR of 5.29 at a dose of 0.75 mg/kg [21]. No adverse events were observed related to the use of MB and no IUI occurred.

### Experimental dyes

Recently, research has focused on novel IV dyes. An overview of the studies and ongoing clinical trials is presented in Table 3.

**Table 3:** Future advancements and ongoing clinical trials

Dye	Article	Reference	Number of animals	Duration of visualization	Doses (IV^a^, mg/kg)	Toxicity in rats (mg/kg)	Ongoing clinical trials
CW800-CA	Tanaka et al. (2007)	[22]	12 rats, 6 pigs	120 min	0.0015–0.015	> 20 [26]	NCT03387410^c^ NCT03106038^c^
	Schols et al. (2014)	[23]	2 pigs		0.007–0.086		
	Korb et al. (2015)	[24]	6 pigs		0.030–0.12		
CW800-BK	Al-Taher et al. (2018)	[25]	3 pigs		0.08–0.3		
(cRGD-)ZW800-1	Verbeek et al. (2014)	[27]	3 rats	< 7.5 h	0.25–30 nmol	> 24.5 [28]	2017-001954-32^e^
Fluorescein	Dip et al. (2014)	[29]	9 rats	< 12 h	7	LD_50_ = 600 [43]	Unknown
	Meershoek et al. (2018)	[30]	3 pigs		5 ml, 100 mg/ml SC/IM^b^		
Liposomal ICG	Portnoy et al. (2015)	[32]	25 mice	> 90 min	8	Liposomes: 10 [34] ICG: LD_50_ = 87 [35]	Unknown
	Friedman-Levi et al. (2018)	[33]	> 12 mice, 2 pigs		4–16		
Genhance 750	Rowe et al. (2012)	[36]	10 swine	> 20 min	0.5	NR	Unknown
UL-766	Cha et al. (2018)	[37]	8 rats	> 60 min	0.09	NR	Unknown
UreterGlow	Mahalingam et al. (2018)	[38]	5 pigs	< 6 h	0.1	NR	Unknown

*ICG* indocyanine green, *NR* not reported

^a^Intravenous injection

^b^Subcutaneous/intramuscular injection

^c^Clinicaltrials.gov database

^d^EU clinical trial register

#### IRDye CW800

Our search produced three animal studies on CW800-CA [22–24] and one on CW800-BK [25] (Table 3). Both dyes were injected IV with low doses in pigs (CW800-CA 0.0015 to 0.12 mg/kg and CW800-BK 0.08 to 0.30 mg/kg). All doses were below the results from initial toxicology reports [26]. The ureters were visualized within 10 min after administration for a total duration of 120 min. In three studies, all ureters could be visualized at any dose [22, 24, 25]. In addition, Tanaka et al. were able to visualize injury and leakage of the ureters [22]. For CW800-CA, the mean SBR was higher than 2 for most doses, with the highest SBR at the lowest dose owing to the extremely low background signals at this dose [24]. For CW800-BK, Al-Taher et al. found SBRs higher than 1 only for the highest dose [25].

IRDye CW800 is currently undergoing clinical translation, with multiple ongoing phase II studies. Two clinical trials focus on the safety and efficacy (NCT03387410), or dosing (NCT03106038) of CW800-BK for ureter visualization (Table 3).

#### ZW800-1

One animal study was identified that tested ureter visualization of cRGD-ZW800-1 in rats [27]. cRGD-ZW800-1 is a targeting integrin conjugated to the fluorophore ZW800-1. This fluorophore (both conjugated and alone) is characterized by near exclusive renal clearance. Low dosages (equivalent to a total human dose of 0.096–1.33 mg) of cRGD-ZW800-1 were injected IV in rats. The study visualized the ureters 10-min post-injection up to a total duration of around 8 h. Toxicity studies of cRGD-ZW800-1 have been performed and report no adverse effects up to doses of 5.0 mg/kg in rats [28]. High SBRs of the ureter were found, peaking around 75.0, which is mostly due to the near absence of background fluorescence. The lowest dose used falls below the Food and Drug Administration (FDA) threshold for microdosing, allowing rapid clinical translation [27].

There is currently one clinical study ongoing that focuses on the use of ZW800-1 for ureter visualization (2017-001954-32, Table 3). Aims of this trial are to assess feasibility and find the optimal dose of ZW800-1.

#### Fluorescein

Our search identified two studies on ureter visualization using fluorescein [29, 30]. The method of administration was by IV injection of fluorescein (7 mg/kg) [29], or subcutaneous and intramuscular injection (5 ml, 100 mg/ml) [30]. The dose is below the median lethal dose of fluorescein in rats (600 mg/kg). Both studies were able to visualize all assessed ureters in rats and pigs. No information was provided on the duration of fluorescence. The studies did not measure SBR but mention subjective excellent visibility [29]. No ongoing clinical trials were found.

#### Liposomal ICG

Portnoy et al. described a liposomal formulation in which ICG is passively absorbed in a liposome [31]. This liposomal encapsulation of ICG increases the renal clearance of ICG, allowing visualization of the ureters. Two animal studies described liposomal ICG for ureter visualization [32, 33]. Portnoy et al. demonstrated that liposomal ICG improved renal excretion compared to free ICG in mice [32], and Friedman-Levi et al. performed a dose-finding study in mice [33]. Both studies injected liposomal ICG IV (8, and 4 to 16 mg/kg, respectively) and visualization of the ureters was achieved 10-min post-injection for a total duration of at least 90 min. Experiments in mice showed mean SBRs higher than 2 at doses above 4 mg/kg. However, relatively bright background from surrounding tissue was observed when 3 mg/kg liposomal ICG was injected in pigs [33]. Toxicity information is not available for liposomal ICG, but for the separate components. Knudsen et al., have observed genotoxic effects of liposomes at concentrations above 10 mg/kg in rats, and the median lethal dose for ICG is 87 mg/kg separately [34, 35]. No ongoing clinical trials were identified.

#### Genhance 750

Rowe et al. described the use of Genhance 750 as a novel method to evaluate ureteropelvic junction obstruction [36]. In this study, the ureteral obstruction was visualized in swine. After IV injection (0.5 mg), fluorescence was seen 1–2 min after injection for at least 15–17 min. The provided images seem to have a low background, but no measurements were performed.

#### Ul-766

One animal study was identified that described the synthesis and initial tests of UL-766 in rats [37]. UL-766 is a cyanine that is exclusively renally cleared and chemically stable. The method of administration was by IV injection of 0.09 mg/kg. As this is a novel dye, no information is available on its toxicity. Cha et al. compared UL-766 to CW800 in rats. UL-766 was able to visualize the ureter, and no background fluorescence was noted. Visualization was possible immediately after injection for at least 60 min. SBRs of the kidney were measured and were generally two times higher than CW800. No ongoing clinical trials were identified.

#### UreterGlow

One study reported on the synthesis of UreterGlow [38]. IV injection (0.1 mg/kg) allows ureter visualization after 15 min for at least 2 h in pigs. No toxicity information is available. The dye is not exclusively renally cleared, but the authors note low background; however, SBR was not measured.

## Discussion

The aim of this systematic review was to describe the literature on fluorescent dyes for intraoperative ureter detection. We have evaluated two clinically available dyes, ICG and MB, and eight experimental dyes. Two experimental dyes, CW800-BK and ZW800-1, are currently undergoing clinical testing for ureteric visualization in phase I trials.

Direct ureteral injection of ICG appears safe, is ready-to-use, does not require systemic exposure, and seems effective. The disadvantages are related to the route of administration which requires expertise, a catheter, and leads to additional surgical time. Therefore, routine use of ICG for ureter identification might be difficult logistically and not cost-effective. In contrast to ICG, MB has the potential to be routinely used in most patients. MB is injected IV, is safe, and detects most of the ureters in the largest patients cohort (*N* = 40) of ureter detection by NIRF so far. Although reported to be safe, MB is contraindicated in patients suffering from renal insufficiency, glucose-6-phosphate dehydrogenase (G6PD) deficiency, and Heinz body anaemia [39]. Furthermore, our results show that using MB is not possible to visualize all ureters. This could be due to the optical properties of MB which are not optimal for high tissue penetration, as MB emits a fluorescence signal near the lower edge of the NIRF range and has low brightness (extinction coefficient × quantum yield). Still, MB is currently preferred for potential routine use in patients with high risk of IUI owing to its simple method of administration. ICG could be used in patients with renal dysfunction or at the surgeon’s discretion.

All experimental dyes can be administered IV and most present SBRs higher than 2, which are considered sufficient [12]. Although the data are limited, CW800, fluorescein, and liposomal ICG do not display much improved performance compared to MB. CW800-CA offers improved brightness, and its spectral properties are higher in the NIRF range compared to MB (Table 1), but the high background signal from surrounding tissues might still interfere with identifying the ureter. CW800-BK is specifically developed for visualizing the ureters, but the initial results seem comparable or even inferior to CW800-CA as lower SBRs are found. However, a recent paper found higher SBRs for CW800-BK in comparison with CW800 and newly developed 800NOS in pigs [40]. Fluorescein has a good safety profile, but its fluorescent emission is within the visible spectrum and thus high autofluorescence and low tissue penetration can be expected. For liposomal ICG, the use of approved components might speed up clinical translation, although its high background noise and commercial unavailability might impede its adaptation. For these reasons, CW800, fluorescein, and liposomal ICG remain suboptimal for ureter visualization.

In contrast, (cRGD-)ZW800-1 has high potential as it allows visualization of the ureter with minimal background noise, high brightness, long duration of visualization, low dosages, and with a potentially rapid clinical translation. More importantly, international communication of the first in human results with ZW800-1 looks promising (presentation at the congress of the European Society of Coloproctology 2018). Newly synthesized dyes UL-766 and UreterGlow might be promising, but costly and laborious regulatory effort still lies ahead before their application in humans. Limited information is available on Genhance 750, and as such benefits of this dye over others are unclear.

Summarizing these results, the definition of an ideal dye for ureter visualization would include exclusive renal clearance, with low background noise from surrounding tissues. In this way, ureteral injection through a catheter or NIRF-lighted stents is not needed for ureter imaging, which in turn entails the possibility of trauma to the ureters and required expertise of the urologist for placement. Fluorescent properties associated with high tissue penetration, such as high brightness and emission in the NIRF range, would facilitate visualization in the majority of patients. The dye should be safe to administer IV, and allowed for ureteral visualization using low doses. Fluorescence should be visible in the ureter shortly after injection for the duration of the entire procedure. In the future, the dye should ideally be able to be used alongside tumour-specific dyes.

We recommend that in order to accelerate the search for the ideal dye for ureter visualization, studies should improve their methodology, and imaging systems optimized for the dye should be used. In addition, studies should focus on collecting clinically relevant information, for example, by identifying the ureter using fluorescence and white light independently. Assessment of the usefulness of fluorescence by surgeons, effects on surgical time, and changes to intraoperative management might also be interesting metrics. Aside from estimating the intensity of the background, the added value of measuring SBR is debatable. Differences between patient groups, imaging settings, region of interest selection, and distance of the laparoscope to the ureter all affect SBR [12]. As a result, these values often cannot be compared between different studies. Translation of SBR from animal models such as the pig is also questionable. The ureteral wall of pigs is both thinner and covered by less fat compared to humans, leading to overestimations of the SBR. Even the smallest layer of fat was found to inhibit signal detection of CW800-BK, which might pose problems for the technique in obese patients [25].

Other reviews have mainly described the possibility of specific dyes to visualize the ureter. Our review has summarized the initial results of all described dyes and judged each on their feasibility. Furthermore, we have provided characteristics of the dyes that allow for comparisons. Limitations of our study are that little data are available, and not all data are of high quality. Risk of bias in animal studies was often judged as unclear, which is a known issue [13]. However, patient cohorts are too small to expect IUI. As such, no conclusions can be drawn on the effectiveness of NIRF imaging in reducing IUI incidence. Altogether, we were able to reach our primary goal of providing an overview of all currently available and experimental dyes for ureter visualization.

## Conclusions

The currently available dyes, ICG and MB, are safe, but suboptimal for routine intraoperative ureter visualization based on the route of administration and optical properties, respectively. Currently, MB has potential to be routinely used for ureter visualization in most patients, but (cRGD-)ZW800-1 holds potential for this role in the future and is currently undergoing clinical translation. In future studies, larger patient cohorts will need to be examined to assess the feasibility of NIRF imaging to reduce the incidence of IUI.

## Electronic supplementary material

Below is the link to the electronic supplementary material.

Supplementary material 1 (DOCX 47 kb)
