# Supplementary material for: Currently available and experimental dyes for intraoperative near-infrared fluorescence imaging of the ureters: a systematic review
Source: Tech Coloproctol. 2019 Apr 27;23(4):305–13. doi: 10.1007/s10151-019-01973-4 (PMC6536635; doi:10.1007/s10151-019-01973-4)
Supplement: Supplementary file 1 — Supplementary material 1 (DOCX 47 kb) [file 10151_2019_1973_MOESM1_ESM.docx]

**Supplementary Data**

**Supplementary table 1.** Overview of the search used in the Embase database.

| **#** | **Searches** | **Results** |
| --- | --- | --- |
| 1 | upper gastrointestinal tract/ or exp esophagus surgery/ or exp stomach tumor/ or exp esophagus tumor/ or (upper gastrointestinal surger* or upper gastrointestinal cancer* or gastric tube* or gastric conduit* or esophagectom* or oesophagectom* or esophageal cancer* or oesophageal cancer* or esophageal neoplasm* or oesophageal neoplasm* or stomach cancer* or stomach neoplasm*).ti,ab,kw. | 240262 |
| 2 | indocyanine green/ or (indocyanine green or ICG).ti,ab,kw. | 18176 |
| 3 | 1 and 2 | 376 |

**Supplementary table 2.** Inclusion and exclusion criteria for the initial article screening

| **Inclusion** | **Exclusion** |
| --- | --- |
| Articles that used fluorescent dyes for surgical assessment of the ureter | Articles visualizing the urethra or other structures of the urinary tract |
| Animal and human studies are both included | Articles solely describing methods other than fluorescent imaging for visualizing the ureter |
|  | Articles in different languages than English, except Dutch |
|  | Articles based solely on video |
|  | Comments |

**Supplementary table 3.** Quality assessment of the human cohort studies using the Newcastle-Ottawa scale. a,b,c, or d indicate the answer given on the corresponding domain.

|  | **[16]** | **[14]** | **[15]** | **[18]** | **[19]** | **[20]** | **[21]** |
| --- | --- | --- | --- | --- | --- | --- | --- |
| 1) Representativeness of the exposed cohort | ◌, c | ◌, d | ◌, c | ●, a | ●, a | ●, b | ●, a |
| 2) Selection of the non exposed cohort | ◌, c | ◌, c | ◌, c | ◌, c | ◌, c | ◌, c | ◌, c |
| 3) Ascertainment of exposure | ●, a | ◌, c | ●, a | ●, a | ●, a | ●, a | ●, a |
| 4) Demonstration that outcome of interest was not present at start of study | ●, a | ●, a | ●, a | ●, a | ●, a | ●, a | ●, a |
| **Selection SCORE:** | ◌◌●● | ◌◌◌● | ◌◌●● | ●◌●● | ●◌●● | ●◌●● | ●◌●● |
| 1) Comparability of cohorts on the basis of the design or analysis | ●, a | ◌, c | ●, a | ●, a | ●, a | ●, a | ●, a |
| **Comparability SCORE:** | ●◌ | ◌◌ | ●◌ | ●◌ | ●◌ | ●◌ | ●◌ |
| 1) Assessment of outcome | ●, b | ◌, d | ●, b | ●, b | ●, b | ●, b | ●, b |
| 2) Was follow-up long enough for outcomes to occur | ●, a | ●, a | ●, a | ●, a | ●, a | ●, a | ●, a |
| 3) Adequacy of follow up of cohorts | ●, a | ◌, d | ●, a | ●, a | ●, a | ●, a | ●, a |
| **Outcome SCORE:** | ●●● | ◌●◌ | ●●● | ●●● | ●●● | ●●● | ●●● |
| **Total SCORE:** | Fair | Poor | Fair | Good | Good | Good | Good |

◌ = no points, ● = one point

**Supplementary table 4.** Quality assessment of the animal studies using the SYRCLE risk of bias tool.

| **Type of bias** | **Domain** | **[37]** | **[38]** | **[17]** | **[32]** | **[33]** | **[22]** | **[23]** | **[24]** | **[25]** | **[27]** | **[29]** | **[30]** | **[36]** |
| --- | --- | --- | --- | --- | --- | --- | --- | --- | --- | --- | --- | --- | --- | --- |
| Selection bias | Sequence generation | 🗸 | 🗸 | ? | ? | ? | ? | 🗸 | ? | 🗸 | ? | 🗸 | ? | 🗸 |
| Selection bias | Baseline characteristics | 🗸 | 🗸 | 🗸 | 🗸 | 🗸 | 🗸 | 🗸 | 🗸 | 🗸 | 🗸 | 🗸 | ? | 🗸 |
| Selection bias | Allocation concealment | ? | 🗸 | ? | 🗸 | ? | ? | ? | ? | ? | ? | 🗸 | ? | 🗸 |
| Performance bias | Random housing | ? | ? | ? | 🗸 | ? | ? | ? | ? | ? | ? | 🗸 | ? | 🗸 |
| Performance bias | Blinding | ? | 🗸 | ? | 🗸 | ? | ? | ? | ? | ? | ? | 🗸 | ? | ? |
| Detection bias | Random outcome assessment | ? | ? | ? | ? | ? | ? | ? | ? | ? | ? | ? | ? | ? |
| Detection bias | Blinding | ? | ? | ? | ? | ? | ? | ? | ? | ? | ? | ? | ? | ? |
| Attrition bias | Incomplete outcome data | 🗸 | 🗸 | 🗸 | 🗸 | 🗸 | 🗸 | 🗸 | 🗸 | 🗸 | 🗸 | 🗸 | 🗸 | 🗸 |
| Reporting bias | Selective outcome reporting | × | × | 🗸 | 🗸 | 🗸 | 🗸 | 🗸 | 🗸 | 🗸 | 🗸 | 🗸 | × | 🗸 |
| Other | Other sources of bias | × | 🗸 | 🗸 | 🗸 | 🗸 | 🗸 | 🗸 | 🗸 | 🗸 | 🗸 | 🗸 | × | 🗸 |

🗸 = low risk of bias, × = high risk of bias, ? = unclear risk of bias
